# Impact of zinc on immunometabolism and its putative role on respiratory diseases

**DOI:** 10.1097/IN9.0000000000000057

**Published:** 2025-03-05

**Authors:** Jonathan H. Yao, Edwin F. Ortega, Alexander Panda

**Affiliations:** 1 Nutritional Immunology Laboratory, Jean Mayer USDA Human Nutrition Research Center on Aging at Tufts University, Boston, MA, USA; 2 Department of Microbiology and Immunology, University of California, San Francisco, CA, USA; 3 Department of Immunology, Tufts University School of Medicine, Boston, MA, USA

**Keywords:** Zinc, immunity, immunometabolism, respiratory disease

## Abstract

Zinc is the second most abundant trace mineral in the human body and plays a critical role in immune cell function and metabolism. Zinc deficiency impairs immune cell function and is associated with increased susceptibility to respiratory diseases, including pneumonia, influenza, and COVID-19. Zinc homeostasis, maintained by numerous zinc transporters and metal-binding proteins (ie, metallothionein), is essential for coordinating immune cell signaling, gene expression, and enzymatic activities in response to respiratory infections. This article highlights the emerging role of zinc in various aspects of immune function, particularly through its influence on cellular metabolism. Given the significant global burden of respiratory diseases, there is a need to identify effective nutritional interventions that could be readily leveraged to prevent and/or mitigate respiratory disease risk, particularly in older adults who are prone to zinc deficiency. However, the immunometabolic mechanisms underlying zinc’s protective effects remain poorly characterized. Future research should focus on elucidating how micronutrients, such as zinc, can support changes in immune cell metabolism in response to infections. Such efforts will help determine how zinc metabolism and zinc intervention strategies may best be leveraged to prevent or mitigate respiratory disease.

## Graphical abstract

**Figure:**
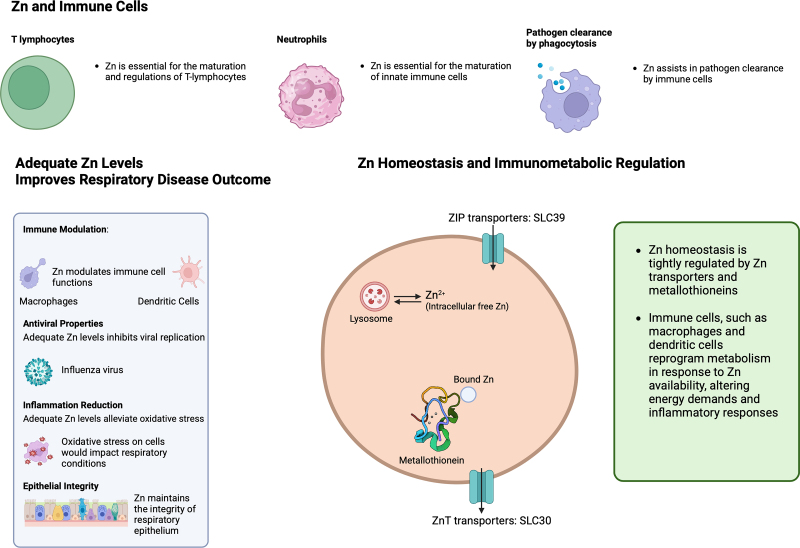

## 1. Introduction

Zinc (Zn) is an essential trace mineral with a pivotal role in human health. Zn is a co-factor for more than 300 enzymes, supporting in catalytic functions, structural integrity, cellular proliferation, differentiation, and protein function ^[1,2]^. Zn also plays a role in gene expression including transcription and translation, primarily mediated by Zn-finger proteins. For example, gene expression of metalloproteinases (MTs) is mediated by Zn bioavailability ^[3]^. Further, Zn possesses immunomodulatory properties. It is required in the development and maturation of both the innate and adaptive immune cells ^[4]^, aids in defending the body against respiratory pathogens ^[5]^, and is needed to maintain the integrity of the epithelial barrier of the human respiratory ^[6]^. Additionally, Zn has been shown to have antimicrobial and antifungal activities ^[7]^. Individuals who are Zn deficient are more susceptible to respiratory diseases due to increased oxidative stress, dysregulated cytokine production, and impaired pathogen clearance ^[8]^. In contrast, Zn supplementation is associated with lower levels of biomarkers of oxidative stress and inflammation in older adults ^[6]^. Taken together, Zn plays a role in central role in protecting the host, directly or indirectly supporting defenses against respiratory pathogens and diseases.

Respiratory diseases remain a significant global public health concern and financial burden. According to the systematic analysis for the Global Burden of Disease Study 2017 ^[9]^, 544.9 million people worldwide had a chronic respiratory disease ^[9]^. The need to identify interventions to mitigate respiratory disease burden has further been emphasized by the COVID-19 pandemic. A comprehensive systematic review and meta-analysis estimated that the total costs of COVID-19 accounted for roughly 86% of health care expenditure and 9.13% of global gross domestic product (GDP) ^[10]^. Similarly, seasonal influenza poses a substantial public health challenge. The World Health Organization reports up to 3 billion influenza cases annually, including 3 to 5 million severe cases ^[11]^ and 5 million hospitalizations globally ^[12]^. In the United States, the Centers for Disease Control and Prevention estimates that seasonal influenza resulted in annual health care costs ranging from $3 billion to $5 billion ^[13]^. Pneumonia remains a significant global health issue as well, responsible for the deaths of approximately 740,180 children under the age of five in 2019, making it the leading infectious cause of death in this age group. In total, this accounted for 14% of all deaths in children <5 years old ^[14]^. An umbrella review focused on Zn status and human disease risk ^[15]^ reported that the incidence and prevalence of pneumonia among the children supplemented with Zn was 13% (relative risk [RR]: 0.87, 95% confidence interval [CI]: 0.81–0.94) and 41% (RR: 0.59, 95% CI: 0.35–0.99) lower, respectively, in comparison to their non-supplemented counterparts. Emerging evidence suggests that Zn may have protective properties against non-communicable respiratory diseases, such as chronic obstructive pulmonary disease (COPD) and asthma ^[5]^. Furthermore, research suggests that Zn may reduce the duration of common cold symptoms by inhibiting the replication of rhinovirus, the common causative agent of the cold ^[16]^.

**Figure 1.**
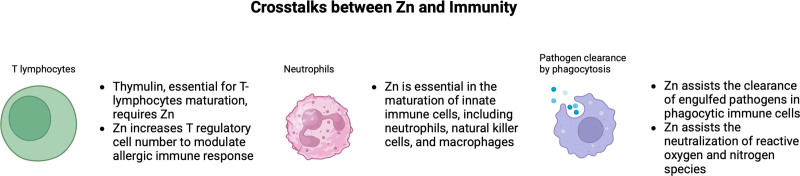
Crosstalks between Zn and immunity.

In this article, we briefly review the role of Zn in immune cell development, function, and response to pathogens (Figure 1), as well as its impact on immune cell metabolism and potential downstream effects on respiratory health and disease risk. We review how Zn status and immune function are impacted by aging, highlighting how Zn supplementation may mitigate immunosenescence and inflammation. We identify demographic groups in the United States that are highly susceptible to Zn deficiency and may benefit the most from targeted supplementation strategies. We discuss future research directions, emphasizing the need for optimizing Zn supplementation doses for large-scale interventions, address knowledge gaps, and areas for further investigation. Overall, we provide information to emphasize the importance of Zn, its emerging role in immunometabolism, and its implications for host immunity and respiratory disease prevention.

## 2. Zinc, immunometabolism, and immune cell function

Recently, the role of cellular metabolism in modulating immune cell function has been increasingly recognized, extending beyond its traditional role of providing energy. Immunometabolism refers to the integration of cellular metabolism with immune cell function. Metabolites and metabolic flux play a critical role in regulating cell signaling and differentiation by influencing the expression, behavior, and specialization of immune cells, ultimately orchestrating immune responses. Different subsets of immune cells adopt unique metabolic programs tailored to their specific needs and microenvironment ^[17]^. For instance, glucose uptake is vital for T cell activation and effector functions, while increased fatty acid oxidation (FAO) is essential for memory T cell longevity ^[18]^.

**Figure 2.**
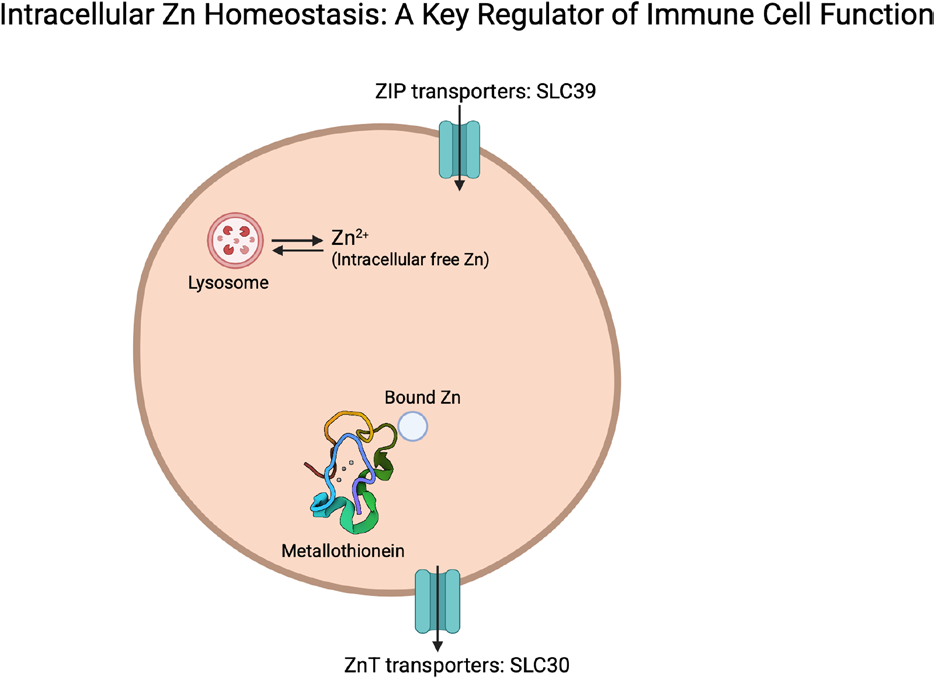
**Intracellular Zinc (Zn) homeostasis: a key regulator of immune cell function.** Zinc is a vital trace element that plays a crucial role in cellular function. Its homeostasis is tightly regulated by a network of Zn transporters, metallothioneins (MTs), and Zn-binding proteins to maintain optimal intracellular concentrations. Lysosomes act as a Zn reservoir and regulate Zn intracellular homeostasis. Zn homeostasis influences immune activation, oxidative stress responses, and pathogen clearance. For example, the Zn importer SL39A7 has been shown to support a pro-inflammatory state in macrophages, necessary for pathogen clearance. Disruptions in Zn homeostasis can lead to immune dysfunction, highlighting its critical role in maintaining immune resilience. MTs, metalloproteinases.

The scope of immunometabolism can be expanded to encompass the role of micronutrients, which are essential for catalyzing metabolism and cellular differentiation through changes in gene expression and signaling pathways. Micronutrients offer the opportunity to fine-tune immunometabolism potentially opening novel therapeutic avenues or preventative strategies. One such micronutrient is Zn. Zn homeostasis is maintained by 24 Zn-transporting proteins and 4 MTs (Figure 2). Zn transporters, including Zn “importers” (SLC39/ZIPs) and “exporters” (SLC30/ZnTs), play a critical role in regulating intracellular Zn levels ^[19,20]^. The SLC30 family includes 10 Zn transporters (ZnT1-10), which reduce cytoplasmic Zn level by exporting it out of the cytoplasm or into intracellular organelles ^[8]^. By targeting specific metabolic processes via Zn, it may be possible to modulate or enhance immune responses.

## 3. Zinc and innate immune cells: macrophages, dendritic cells, and neutrophils

Macrophages (Mφ) are innate immune cells critical for pathogen clearance and homeostasis. As one of the most abundant immune cells in the lung, their phagocytic ability is essential for responding to respiratory infections ^[21]^. Lung Mφ consist of two distinct populations: alveolar Mφ, which serve as first-line defenders against infiltrating respiratory pathogens, and interstitial Mφ, located in the lung interstitial and regulate innate immune responses ^[22]^.

Zn homeostasis plays a pivotal role in Mφ function. The Zn importer SLC39A10 has been found to regulate intracellular Zn levels in response to lipopolysaccharide (LPS) stimulation ^[23]^ in vivo. Dysregulated Zn homeostasis impairs Mφ function, preventing phagocytic uptake of parasites ^[24]^. For instance, an in vivo study with ZIP1 knockout mice reported a reduction in efferocytosis in alveolar Mφ due to impaired Zn uptake ^[25]^. Zn also influences Mφ inflammatory signaling by modulating key signaling pathways. It sustains nuclear factor kappa B (NF-κB) activation by preventing dephosphorylation and enhances mitogen-activated protein kinases (MAPK) signaling (eg, p38, MEK1/2, ERK1/2), thereby promoting the production of pro-inflammatory cytokines such as TNF-α, IL-1β, and IL-6. Conversely, Zn attenuates inflammation by upregulating inhibitors like A20, which suppress NF-κB activity ^[19,26]^. Zn deficiency can lead to disruption of immune homeostasis, depending on long-term or short-term deficiency. Long-term Zn deficiency compromises lysosomal integrity, activating the NLRP3 inflammasome and increasing IL-1β secretion in vivo ^[26,27]^. Short-term effects inhibit inflammatory activation, reducing IL-6 and TNF-α productions, as observed in human monocytes in vitro ^[28]^. Zn is also essential for the activity of classical zinc-dependent histone deacetylases, which mediate lysine deacetylation. This process reprograms Mφ metabolism to regulate an inflammatory response ^[29]^.

Mφ adapt their functional state in response to environment cues. In vitro, Mφ can take a pro-inflammatory (M1) or anti-inflammatory (M2) state, each requiring distinct metabolic states ^[17]^. M1 Mφ rely on glycolysis to produce inflammatory cytokines and reactive oxygen species (ROS), which is facilitated by the stabilization of hypoxia inducible factor 1α (HIF1α), and the activation of glycolytic genes including *GLUT1* ^[30]^. In M1 Mφ, the Krebs cycle is interrupted at two points. First, the conversion from citrate to isocitrate is interrupted, which leads to the accumulation of citrate in the cytoplasm, promoting the production of nitric oxide. Second, the disruption of aconitate to isocitrate conversion by aconitase leads to itaconate accumulation. Itaconate inhibits succinate dehydrogenase, leading to the accumulation of succinate, inducing the activation of HIF-1α, which promotes the generation of pro-inflammatory cytokine IL-1β ^[30]^. In contrast, M2 Mφ depend on oxidative phosphorylation (OXPHOS) and FAO. M2 Mφ maintain an intact Krebs cycle, which provides substrates such as NADH and FADH2 for the electron transport chain, which supports energy production ^[30]^. ZIPs are crucial in regulating Mφ activation states and phagocytosis. In an in vitro study ^[31]^, SLC39A7 knockdown in THP-1-derived Mφ impaired phagocytosis but could be restored with Zn supplementation. Deficiency in the SLC39A7 gene leads to decreased intracellular Zn levels, skewing Mφ toward the anti-inflammatory M2 state, which suggests that SLC39A7 maintains the pro-inflammatory responses necessary for pathogen clearance. Further, the in vitro THP-1 cell line also suggested that SLC39A8 (ZIP8) regulates pro-inflammatory responses by acting as a direct transcriptional target of NF-кB, which leads to the downstream downregulation of IкB kinase (IKK) activity via Zn ^[32]^. Similarly, a study found that in primary Mφ isolated from human blood, LPS upregulated SLC39A14 (ZIP14) ^[33]^. The induction was highly dependent on calcium binding, guanine cytosine (GC)-rich DNA binding, and NF-кB downregulation ^[33]^. An in vitro study with primary Mφ demonstrated that ZIP14 expression is significantly upregulated by both IL-6 and LPS. These findings highlight ZIP14 as a critical Zn transporter and suggest a pivotal role in regulating Mφ function and activation states ^[33]^. By modulating inflammation and immune responses through the regulation of Zn transporters, we may be able to support or enhance a discrete Mφ functional state.

Dendritic cells (DCs) are a group of hematopoietic cells that recognize infectious pathogens and commensals ^[34]^ through pattern recognition receptors (PRRs). PRR engagement initiates signaling pathways that induce cellular activation through changes in gene expressions ^[35]^. Activated DCs migrate to the lymph nodes and present antigens to memory and naive T and B cells, initiating an adaptive immune response ^[35]^. Different subsets of DCs include conventional DCs (cDCs), monocyte-derived DCs, plasmacytoid DCs (pDCs), and Langerhans cells, each adopting a distinct metabolic profile. cDCs in the respiratory epithelium primarily rely on OXPHOS and FAO. The mammalian target of rapamycin (mTOR) signaling pathway regulates these metabolic pathways by modulating transcription factors involved in lipid and mitochondrial metabolism, such as the myelocytomatosis viral oncogene homolog (MYC), as shown in vivo ^[36]^. On the other hand, pDCs predominantly depend on OXPHOS, enabling high production of type 1 interferons in response to viral infections ^[34]^. In an in vitro study, activation of DCs through TLR-4 leads to transient increases in glycolysis ^[37]^ to support cytokine production and antigen presentation. However, limited information exists regarding the role of trace metals in DC metabolism. Zn appears to be crucial for DC function, influencing their maturation, antigen presentation capability, and inflammatory responses. For example, LPS-induced TLR-4 signaling alters Zn transporter expression in DCs in vitro ^[38]^, partly via the TRIF-TLR signaling pathway ^[38]^. This ex vivo study demonstrated that treatment of bone marrow–derived DCs with LPS altered the expression of ZIP6, accompanied by the upregulation of major histocompatibility complex (MHC) class II and costimulatory molecules, CD86 ^[38]^. Since MHC class II and CD86 are both critical for T cell activation, any alteration in their function consequently affects adaptive immunity, which relies heavily on antigen presentation and T cell activation. Zn has been shown to influence DC maturation by inducing a tolerogenic phenotype. This effect is mediated by reducing surface MHC class II expression and promoting tolerogenic markers, including programmed death-ligands (PD-L)1 and PD-L2, as well as the tryptophan-degrading enzyme IDO, in mouse bone marrow–derived ex vivo DCs. These changes were assessed ex vivo using fluorescent-activated cell sorting ^[39]^. Fluorescent-activated cell sorting was used to distinguish DC populations with high vs low MHC class II expression following Zn exposure. PD-L1 and PD-L2 expression were also evaluated in these DC subsets, with or without Zn. Flow cytometric analysis of MHC class II- and PD-L1/2-expressing DCs was determined in both uninfected or yeast-infected DCs.

Zn is also essential for neutrophil function. Chelation of Zn has been shown to reduce neutrophil chemotaxis ^[40]^. Similarly, Zn-deficient status impacts phagocytosis by impairing ROS production/oxidative burst and cytokine production, as was shown with *Streptococcus pneumoniae* ^[41]^ in vitro. Additionally, Zn homeostasis is vital for neutrophil extracellular trap formation, as both excess and deficient Zn status inhibit superoxide production in neutrophils, directly disrupting neutrophil extracellular trap formation as shown in ex vivo studies using isolated human neutrophils ^[42]^. In vitro studies have also demonstrated that Zn induces multimerization of the killer-cell immunoglobulin receptors in human natural killer (NK) cells, which controls the activation of NK cells and plays an important role in recognizing MHC class I molecules ^[43]^. Zn metabolism in neutrophils and NK cells may be crucial for their function, but much more mechanistic evidence is needed.

Finally, Zn deficiency is linked with elevated levels of pro-inflammatory mediators, increased ROS, and heightened susceptibility to severe inflammatory responses and respiratory diseases. Clinical studies in older adults suggest that Zn supplementation can alleviate these effects ^[44]^. ROS is a double-edged sword, while low to moderate levels of ROS are essential for normal cellular signaling and immune defense, excessive production can lead to oxidative stress and contribute to chronic inflammation and tissue damage. Higher ROS production has been associated with Zn deficiency ^[45]^, and indicated by low levels of glutathione and superoxide dismutase (SOD) in a cross-sectional study ^[46]^. Another cross-sectional study associated low levels of plasma Zn in type 2 diabetic patients with elevated response to oxidative stress and reduced levels of SOD ^[47]^. Zn acts as a co-factor for SOD, which converts harmful superoxide radicals into hydrogen peroxide and oxygen, thereby reducing cytotoxicity. Zn stabilizes cell membranes and protects them from oxidative damage by inhibiting nicotinamide adenine dinucleotide phosphate (NADPH) oxidase, an enzyme that generates ROS. Additionally, Zn induces the synthesis of MTs, proteins that bind and sequester ROS ^[45]^. MTs also function as Zn reservoirs, mediators of detoxification, and regulators of redox balance.

In summary, Zn is required for the development, maturation, differentiation, and the effective functions of innate immune cells including neutrophils, macrophages, and NK cells. The phagocytic activity of the first-line defending cells, such as neutrophils and macrophages, is highly dependent on Zn to alter their metabolism in response to extracellular stimuli. By mediating changes in pro-inflammatory or anti-inflammatory status, host and cellular Zn status effectively influences the metabolic phenotype of innate immune cells.

## 4. Zinc and adaptive immune cells: T and B cells

T and B cells are central to adaptive immunity, serving as the primary components of cell-mediated and humoral immunity, respectively. These adaptive immune cells exist in various compartments in the lung ^[48]^. Zn influences the maturation and differentiation of the T cells. Further, the activity of thymulin, a Zn-dependent hormone, is involved in T cell differentiation in the thymus. Thymulin levels are disturbed in a Zn-deficient state by resulting in a decreased level of thymulin in the serum, which affects immune responses by altering T cell subpopulations and reducing T cell-mediated immunity ^[49]^.

Zn status impacts T cells differentiation and function. Zn supplementation augments the TGF-β1-dependent induction pathway of T-regulatory cells through the transcription factor Foxp3 ^[50]^. Zn deficiency is associated with disturbances in the metabolism of cytotoxic T cells, including a lower frequency of CD73^+^ (Ecto-5’-nucleotidase) expression, which is involved in the purinergic signaling pathway ^[51]^. Activated cytotoxic T cells rely on glycolysis to meet their high energy demands, with Zn serving as a co-factor for lactate dehydrogenase, essential for rapidly proliferating T cells ^[52]^. On the other hand, excessive Zn, that is, ≥100 μM, impairs CD4^+^ T cell function in vitro, specifically, it inhibits glycolysis and OXPHOS, thereby inhibiting activation and proliferation ^[53]^. In allergies, Zn deficiency results in a T_H_1 and T_H_2 functional imbalance, favoring a T_H_2-driven allergic state, while also promoting T_H_17 differentiation and IL-1β expression in vitro ^[54]^. However, excess Zn concentrations, that is, above 100 μM, impair the metabolic fitness of CD4^+^ T cells, by blunting the nuclear expression of the transcription factor MYC in vitro ^[53]^, preventing T_H_1 and T_H_2 differentiation. In vivo, the presence of high Zn concentrations (100 μM) has also been shown to impair T_H_1 CNS autoimmunity and T_H_2 asthmatic airway inflammation ^[53]^.

Zn is also essential for B cell development and function. Zn influences B cell receptor (BCR) activation, affecting kinases like protein kinase C (PKC) and MAPK, and transcription factors such as nuclear factor of activated T cells (NFAT) and NF-кB as shown in vivo ^[55]^. Acute and chronic Zn deficiency have contrasting effects: acute Zn deficiency results in a more pronounced reduction in B cells compared with chronic Zn deficiency, suggesting an adaptive response over time ^[56]^. However, excessive Zn has been shown to promote the development and survival of B cells through the regulation of ZIP10 by STAT3 and STAT5 in vivo ^[57]^. Notably, STAT3 is widely recognized in the proliferation, survival, and invasion of various human malignancies, including B cell lymphoma ^[57]^. This regulatory pathway is crucial for suppressing apoptosis of human B cells in lymphoma ^[8]^. Interestingly, in the early developmental stage, B cells are highly susceptible to apoptosis in a Zn-deficient state. A similar observation in T cells is observed, fewer naive T are found under Zn deficiency ^[58]^. Thus, maintaining Zn homeostasis is essential for the proper functioning and development of B cells. Zn also plays a direct role in BCR-induced signaling; Cre-transgenic conditional ZIP10 knockout in vivo studies in B cells demonstrated severe disruptions in signaling and immunoglobulin (Ig) production ^[59]^. Metabolic reprogramming is essential for B cell activation and Ig production. Upon activation, B cells increase both glycolysis and OXPHOS to meet their energy and biosynthetic demands, with a significant upregulation of the glucose transporter GLUT1 in vivo ^[60]^. Further, control B cells show balanced metabolic increases, whereas anergic B cells exhibit minimal metabolic activity and reduced Ig production. In contrast, B cells chronically exposed to B cell activating factor show exaggerated metabolic responses and increased Ig production ^[60]^.

Taken together, these findings highlight Zn’s role in B and T cell metabolism, which can impact signaling, development, and survival, thus emphasizing its importance in adaptive immune cell function ^[8]^.

## 5. Zinc and respiratory diseases

**Figure 3.**
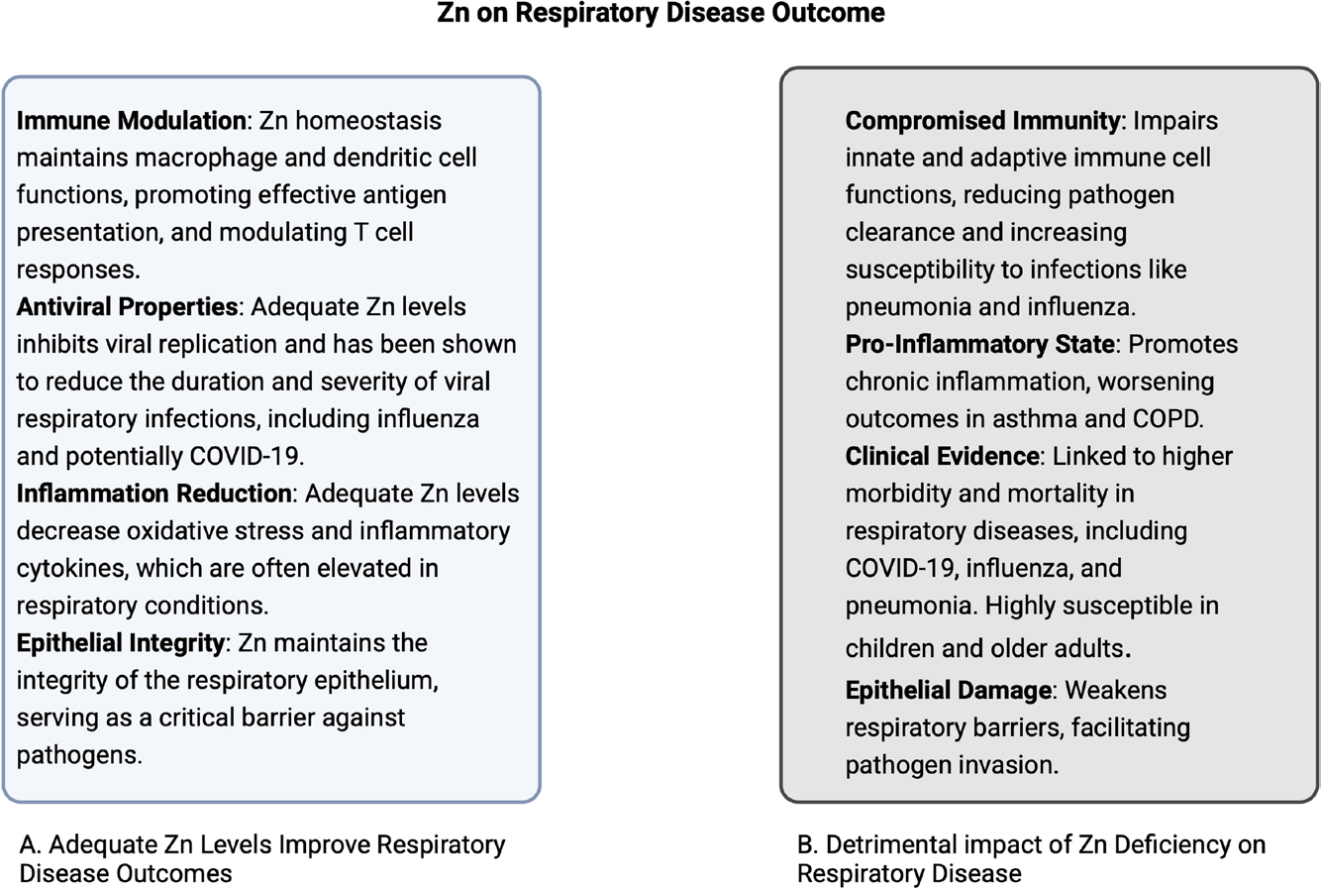
Zn on respiratory disease outcome.

Given Zn’s pivotal role in immune cell function, the impact of Zn status has been studied in the context of preventing and treating respiratory infections, including the common cold, flu, pneumonia, and COVID-19 (Figure 3).

One review highlighted Zn’s impact on lung immune cells and its role in reducing viral titer following influenza infection ^[61]^. The anti-viral effect of Zn appears to be virus specific. Zn inhibits viral replication by inhibiting the viral polymerases following infection by influenza. Additionally, Zn-finger protein ZFP36L1 attenuates the production of viral proteins, matrix protein, hemagglutinin, and neuraminidase by targeting their RNA transcripts in vitro ^[62]^. Increases in the “labile” Zn pool of RSV-infected cells coincided with the induction of ROS. Moreover, depletion of the intracellular Zn pool and additional exogenous ROS is associated with enhanced RSV infection in vitro ^[63]^. In both in vitro and in vivo experiments, Zn homeostasis has been found to exert anti-viral activity, showcasing its potential as a therapeutic agent ^[64]^.

Most recently, from December 2019 to November 2024, more than 700 million cases of COVID-19 were reported, with more than 7 million confirmed deaths ^[65]^. A systematic review and meta-analysis (5 studies, 1506 patients) suggested that Zn supplementation may lower mortality in COVID-19 patients ^[64]^, though no dose-response effect was evaluated. Another meta-analysis (11 studies) found a moderate association between higher Zn levels and less severe disease (standardized mean difference [SMD]: 0.50; 95% CI: 0.32–0.68). However, a separate meta-analysis of 3 studies showed no correlation between serum Zn levels and mortality (SMD: 1.66, 95% CI: −1.42–4.47). While promising, the impact of Zn on COVID-19 is inconclusive ^[66]^. At the molecular level, Zn cations (Zn^2+^) are shown to inhibit the replication of SARS-coronavirus RNA polymerase ^[67]^. Zn also plays a role in the viral entry of severe acute respiratory syndrome coronavirus 2 (SARS-CoV-2) by the angiotensin-converting enzyme 2 (ACE2) function, which facilitates the entry of the virus into the host cell via the spike protein. ACE2, as a metallopeptidase, requires Zn for metabolizing its substrates, but excess Zn (>10 µM) inhibits ACE2 activity leading to higher levels of angiotensin 2 and promoting inflammation ^[68]^. It is suggested that 10 µM represents the upper limit for optimal ACE2 activity. Human plasma/serum Zn levels typically range from 12 to 17 µM ^[69]^, placing 10 µM at the lower physiological spectrum. In human lung cell lines, while Zn alone does not significantly alter ACE2 expression, its combination with NF-кB inhibitors, such as triclabendazole or emetine, suppresses ACE2 in a dose-dependent manner. These findings highlight the potential of Zn supplementation as part of therapeutic strategies, particularly in combination with NF-кB inhibitors ^[70]^.

Zn also plays a vital role in non-communicable respiratory diseases. For example, in COPD ^[25]^, Zn contributes to the pathogen clearance function of alveolar macrophages ex vivo ^[25]^. In COPD, impaired clearance of apoptotic epithelial cells by alveolar macrophages was attributed to impaired efferocytosis due to Zn deficiency. Efferocytosis involves a unique metabolic reprogramming and is characterized by the upregulation of specific members of the solute carrier family of membrane transport proteins – essential for glucose uptake and lactate release ^[16]^. In a BALB/c mice study, dietary Zn restriction activates caspase-3 and leads to the accumulation of apoptotic epithelial cells in the bronchioles, exacerbating airway inflammation ^[71]^. Zn transporters ZIP1 and ZIP2 respond differently during Zn deficiency. ZIP1 facilitates Zn uptake during acute deficiency, while ZIP2 is involved in Zn redistribution to cells that need it most during prolonged deficiency ^[72,73]^.

Asthma is a chronic respiratory condition characterized by a hyper-responsiveness of the immune system to environmental or common allergens. T_H_2 cells play a central role in orchestrating the immune response in asthma. They produce IL-5, IL-4, and IL-13, which activate eosinophils and contribute to airway inflammation and hyper-responsiveness. In both in vitro and in vivo experiments, Zn supplementation relieved asthmatic symptoms. Using transcriptomic analysis, Krone et al ^[53]^ demonstrated in vitro that a therapeutic infusion solution containing Zn aspartate inhibited T_H_2 airway inflammation by altering CD4^+^ T cell function and viability in a dose-dependent manner. Specifically, the presence of Zn downregulated the expression of cell cycle, glycolytic, and tricarboxylic acid cycle genes, which results in reduced glycolysis, OXPHOS, metabolic fitness, and viability. Additionally, high Zn concentrations stabilize FOXO1 expression, a negative regulator of T cell metabolism, proliferation, and effector function. These findings suggest that maintaining Zn homeostasis could offer therapeutic benefits by reducing T_H_2-mediated airway inflammation in asthma.

## 6. Zinc and aging

Older adults are particularly vulnerable to Zn deficiency due to suboptimal micronutrient intake, often caused by limited access to healthy food, impaired absorption due to age-related defects in nutrient uptake, diminished sensory functions ^[74]^, and an overall decline in health status ^[75]^. Older adults aged greater than 60 years have Zn intakes below 50% of the recommended dietary allowance. Age-related Zn deficiency is highly associated with the dysfunction of intracellular Zn signaling via the Zn transporters. With aging, ZIP1 and 2 gene expression is downregulated in peripheral blood mononuclear cells ^[76]^. Additionally, MTs are elevated with older age which reduces availability of intracellular Zn ^[75]^. Low Zn intake among older adults may also increase plasma levels of ZIP1 and 3, which may be caused by decreasing the endocytosis rate in response to decreasing ZIP2 expression ^[77]^. Impaired transporter protein function is highly correlated with aging ^[78]^. Interestingly, over-expression of IL-6 and TNF-α mRNA is provoked by marginal Zn deficiency in both young and older adults, but the compensatory effect of intracellular Zn increment is not as effective in older adults ^[79]^. Zn deficiency in older individuals also contributes to immunosenescence, a progressive deterioration of the immune system associated with aging. This condition involves reduced thymus activity, a shift in T helper cell activity toward T_H_2, and impaired function of innate immune cells like phagocytes and NK cells ^[33]^. Despite numerous trials on Zn supplementation for older adults, results have been inconsistent due to different intervention strategies, doses, or forms of Zn supplementation ^[80]^. Additionally, consideration of genetic polymorphisms may be required. Zn supplementation may be more effective for older adults with a specific IL-6 polymorphism ^[44]^. Interestingly, dietary changes, such as adopting the Mediterranean diet, may ameliorate Zn deficiency and its downstream effects on the immune response ^[44]^.

## 7. Zinc intake in the United States

In the United States, dietary sources of Zn include meat, seafood, dairy products, nuts, and legumes. The recommended daily intake varies by age, gender, and life stage. The latest data on Zn intake among adults in the United States indicate that most Americans consume adequate amount of Zn; however, certain groups of the population have marginal intakes such as the elderly, vegetarians, and individuals with malabsorptive conditions ^[81]^. Factors influencing Zn status include dietary habits ^[82]^, bioavailability of Zn from different food sources ^[83]^, and interactions with other nutrients ^[83]^, particularly copper. Strategies to optimize Zn intake involve dietary diversification, fortification, and supplementation. Tailoring Zn supplementation strategies to specific demographics and health statuses can improve overall nutrient balance and health outcomes.

## 8. Conclusions and future directions

Zn plays a pivotal role in immunity contributing to respiratory disease risk by influencing various aspects of immune cell function in part to its impact on gene expression, protein activity, and cell signaling ^[15]^. Recent findings implicate immunometabolism as a key aspect of immune cell differentiation and function. However, to date, the focus has been on metabolites or substrates that fuel metabolism, while micronutrients (ie, Zn, iron, vitamin D, NAD+), which support cellular metabolism, have received less attention. Herein we provide a brief review on the role of Zn in supporting metabolic function by acting as an enzyme co-factor and participating in gene expression as well as redox balance. Adequate levels of Zn ensure that cells can respond to metabolic needs due to changing immune demands. The ability to respond to stimuli depends on the differential expression of ZIPs and ZnTs. The current literature suggests that Zn supplementation for those who are Zn deficient could potentially be leveraged to support innate and adaptive immune cell responses. Identifying those most at risk for Zn deficiency can significantly fast track our ability to intervene with dietary interventions or tailored supplementation strategies. Regular dietary assessments and possibly plasma Zn level measurements can help identify individuals at risk of deficiency. It is essential to introduce Zn-rich foods such as meat, seafood, dairy products, nuts, and legumes into the diets of patients with Zn deficiency. For those unable to meet their Zn needs through diet alone, Zn supplementation may be necessary. However, excessive Zn may also tip the balance of Zn homeostasis unfavorably. Further, different disease states might require different Zn supplementation dosages and strategies. Importantly, to date, no studies have reported the correlation between blood levels of Zn and both total and labile cellular Zn levels. Therefore, it is unclear whether increases in serum Zn levels lead to parallel increases in immune cell Zn levels. Overall, a proactive and informed approach by health care professionals could be crucial in preventing Zn deficiency and optimizing patient health. However, further research is needed to guide these decisions effectively.

## Conflicts of interest

The authors declare that they have no conflicts of interest.

## Funding

This work was supported by the National Institutes of Health 5R34HL153277-02 Zinc intervention in the prevention of pneumonia in elderly. We declare that the supporting source had no such involvement or restrictions regarding publication.

## References

[1] Rink L Gabriel P. Zinc and the immune system. Proc Nutr Soc. 2000;59(4):541-52. 11115789 10.1017/s0029665100000781

[2] Chasapis CT Loutsidou AC Spiliopoulou CA. Zinc and human health: an update. Arch Toxicol. 2012;86(4):521-34. 22071549 10.1007/s00204-011-0775-1

[3] Mocchegiani E Muzzioli M Giacconi R. Zinc, metallothioneins, immune responses, survival and ageing. Biogerontology. 2000;1(2):133-43. 11707929 10.1023/a:1010095930854

[4] Gammoh NZ Rink L. Zinc and the immune system. In: Mahmoudi M Rezaei N, eds. Nutrition and Immunity. Springer International Publishing; 2019. p. 127-58.

[5] Luan R Ding D Xue Q. Protective role of zinc in the pathogenesis of respiratory diseases. Eur J Clin Nutr. 2023;77(4):427-35. 35982216 10.1038/s41430-022-01191-6 PMC9387421

[6] Prasad AS. Zinc is an antioxidant and anti-inflammatory agent: its role in human health. Front Nutr. 2014;1:14. 25988117 10.3389/fnut.2014.00014 PMC4429650

[7] Pasquet J Chevalier Y Pelletier J. The contribution of zinc ions to the antimicrobial activity of zinc oxide. Colloids Surf A. 2014;457:263-74.

[8] Wessels I Maywald M Rink L. Zinc as a gatekeeper of immune function. Nutrients. 2017;9(12):1286. 29186856 10.3390/nu9121286 PMC5748737

[9] Collaborators GCRD. Prevalence and attributable health burden of chronic respiratory diseases, 1990-2017: a systematic analysis for the Global Burden of Disease Study 2017. Lancet Respir Med. 2020;8(6):585-96. 32526187 10.1016/S2213-2600(20)30105-3 PMC7284317

[10] Faramarzi A Norouzi S Dehdarirad H. The global economic burden of COVID-19 disease: a comprehensive systematic review and meta-analysis. Syst Rev. 2024;13(1):68. 38365735 10.1186/s13643-024-02476-6 PMC10870589

[11] World Health Organization. Influenza (seasonal). Accessed July 3, 2024. https://www.who.int/news-room/fact-sheets/detail/influenza-(seasonal)

[12] Lafond KE Porter RM Whaley MJ; Global Respiratory Hospitalizations–Influenza Proportion Positive (GRIPP) Working Group. Global burden of influenza-associated lower respiratory tract infections and hospitalizations among adults: a systematic review and meta-analysis. PLoS Med. 2021;18(3):e1003550. 33647033 10.1371/journal.pmed.1003550 PMC7959367

[13] Centers for Disease Control and Prevention. Key facts about influenza (flu). Accessed June 8, 2024. https://www.cdc.gov/flu/about/

[14] World Health Organization. Pneumonia. Accessed July 10, 2024. https://www.who.int/news-room/fact-sheets/detail/pneumonia

[15] Li J Cao D Huang Y. Zinc intakes and health outcomes: an umbrella review. Front Nutr. 2022;9:798078. 35211497 10.3389/fnut.2022.798078 PMC8861317

[16] Morioka S Perry JSA Raymond MH. Efferocytosis induces a novel SLC program to promote glucose uptake and lactate release. Nature. 2018;563(7733):714-8. 30464343 10.1038/s41586-018-0735-5 PMC6331005

[17] Makowski L Chaib M Rathmell JC. Immunometabolism: from basic mechanisms to translation. Immunol Rev. 2020;295(1):5-14. 32320073 10.1111/imr.12858 PMC8056251

[18] Chapman NM Chi H. Metabolic adaptation of lymphocytes in immunity and disease. Immunity. 2022;55(1):14-30. 35021054 10.1016/j.immuni.2021.12.012 PMC8842882

[19] Gao H Dai W Zhao L. The role of zinc and zinc homeostasis in macrophage function. J Immunol Res. 2018;2018:6872621. 30622979 10.1155/2018/6872621 PMC6304900

[20] Na-Phatthalung P Min J Wang F. Macrophage-mediated defensive mechanisms involving zinc homeostasis in bacterial infection. Infect Microbes Dis. 2021;3(4):175-82.

[21] Healy C Munoz-Wolf N Strydom J. Nutritional immunity: the impact of metals on lung immune cells and the airway microbiome during chronic respiratory disease. Respir Res. 2021;22(1):133. 33926483 10.1186/s12931-021-01722-y PMC8082489

[22] Liegeois M Legrand C Desmet CJ. The interstitial macrophage: a long-neglected piece in the puzzle of lung immunity. Cell Immunol. 2018;330:91-6. 29458975 10.1016/j.cellimm.2018.02.001

[23] Gao H Zhao L Wang H. Metal transporter Slc39a10 regulates susceptibility to inflammatory stimuli by controlling macrophage survival. Proc Natl Acad Sci U S A. 2017;114(49):12940-5. 29180421 10.1073/pnas.1708018114 PMC5724256

[24] Wirth JJ Fraker PJ Kierszenbaum F. Zinc requirement for macrophage function: effect of zinc deficiency on uptake and killing of a protozoan parasite. Immunology. 1989;68(1):114-9. 2680908 PMC1385514

[25] Hamon R Homan CC Tran HB. Zinc and zinc transporters in macrophages and their roles in efferocytosis in COPD. PLoS One. 2014;9(10):e110056. 25350745 10.1371/journal.pone.0110056 PMC4211649

[26] Brieger A Rink L Haase H. Differential regulation of TLR-dependent MyD88 and TRIF signaling pathways by free zinc ions. J Immunol. 2013;191(4):1808-17. 23863901 10.4049/jimmunol.1301261

[27] Muroi M Tanamoto K-I. Zinc- and oxidative property-dependent degradation of pro-caspase-1 and NLRP3 by ziram in mouse macrophages. Toxicol Lett. 2015;235(3):199-205. 25929180 10.1016/j.toxlet.2015.04.012

[28] Mayer LS Uciechowski P Meyer S. Differential impact of zinc deficiency on phagocytosis, oxidative burst, and production of pro-inflammatory cytokines by human monocytes. Metallomics. 2014;6(7):1288-95. 24823619 10.1039/c4mt00051j

[29] Shakespear MR Iyer A Cheng CY. Lysine deacetylases and regulated glycolysis in macrophages. Trends Immunol. 2018;39(6):473-88. 29567326 10.1016/j.it.2018.02.009

[30] Viola A Munari F Sánchez-Rodríguez R. The metabolic signature of macrophage responses. Review. Front Immunol. 2019;10:1462. 31333642 10.3389/fimmu.2019.01462 PMC6618143

[31] Xie W Xue Q Niu L. Zinc transporter SLC39A7 relieves zinc deficiency to suppress alternative macrophage activation and impairment of phagocytosis. PLoS One. 2020;15(7):e0235776. 32645059 10.1371/journal.pone.0235776 PMC7347223

[32] Liu MJ Bao S Gálvez-Peralta M. ZIP8 regulates host defense through zinc-mediated inhibition of NF-κB. Cell Rep. 2013;3(2):386-400. 23403290 10.1016/j.celrep.2013.01.009 PMC3615478

[33] Sayadi A Nguyen AT Bard FA. Zip14 expression induced by lipopolysaccharides in macrophages attenuates inflammatory response. Inflamm Res. 2013;62(2):133-43. 23052185 10.1007/s00011-012-0559-y

[34] Pearce EJ Everts B. Dendritic cell metabolism. Nat Rev Immunol. 2015;15(1):18-29. 25534620 10.1038/nri3771 PMC4495583

[35] Kawai T Akira S. Toll-like receptors and their crosstalk with other innate receptors in infection and immunity. Immunity. 2011;34(5):637-50. 21616434 10.1016/j.immuni.2011.05.006

[36] Wang R Dillon CP Shi LZ. The transcription factor Myc controls metabolic reprogramming upon T lymphocyte activation. Immunity. 2011;35(6):871-82. 22195744 10.1016/j.immuni.2011.09.021 PMC3248798

[37] Everts B Amiel E Huang SC. TLR-driven early glycolytic reprogramming via the kinases TBK1-IKKɛ supports the anabolic demands of dendritic cell activation. Nat Immunol. 2014;15(4):323-32. 24562310 10.1038/ni.2833 PMC4358322

[38] Kitamura H Morikawa H Kamon H. Toll-like receptor-mediated regulation of zinc homeostasis influences dendritic cell function. Nat Immunol. 2006;7(9):971-7. 16892068 10.1038/ni1373

[39] George MM Subramanian Vignesh K Landero Figueroa JA. Zinc induces dendritic cell tolerogenic phenotype and skews regulatory T cell-Th17 balance. J Immunol. 2016;197(5):1864-76. 27465530 10.4049/jimmunol.1600410 PMC4992588

[40] Hasan R Rink L Haase H. Chelation of free Zn²^+^ impairs chemotaxis, phagocytosis, oxidative burst, degranulation, and cytokine production by neutrophil granulocytes. Biol Trace Elem Res. 2016;171(1):79-88. 26400651 10.1007/s12011-015-0515-0

[41] Herring SE Mao S Bhalla M. Mitochondrial ROS production by neutrophils is required for host antimicrobial function against *Streptococcus pneumoniae* and is controlled by A2B adenosine receptor signaling. PLoS Pathog. 2022;18(11):e1010700. 36374941 10.1371/journal.ppat.1010700 PMC9704767

[42] Freitas M Porto G Lima JL. Zinc activates neutrophils’ oxidative burst. Biometals. 2010;23(1):31-41. 19760108 10.1007/s10534-009-9264-x

[43] Kumar S Rajagopalan S Sarkar P. Zinc-induced polymerization of killer-cell Ig-like receptor into filaments promotes its inhibitory function at cytotoxic immunological synapses. Mol Cell. 2016;62(1):21-33. 27058785 10.1016/j.molcel.2016.03.009 PMC4826557

[44] Mocchegiani E Romeo J Malavolta M. Zinc: dietary intake and impact of supplementation on immune function in elderly. Age (Dordr). 2013;35(3):839-60. 22222917 10.1007/s11357-011-9377-3 PMC3636409

[45] Marreiro DD Cruz KJ Morais JB. Zinc and oxidative stress: current mechanisms. Antioxidants (Basel). 2017;6(2):24. 28353636 10.3390/antiox6020024 PMC5488004

[46] Habib SA Saad EA Elsharkawy AA. Pro-inflammatory adipocytokines, oxidative stress, insulin, Zn and Cu: interrelations with obesity in Egyptian non-diabetic obese children and adolescents. Adv Med Sci. 2015;60(2):179-85. 25827128 10.1016/j.advms.2015.02.002

[47] Lima VB Sampaio FA Bezerra DL. Parameters of glycemic control and their relationship with zinc concentrations in blood and with superoxide dismutase enzyme activity in type 2 diabetes patients. Arq Bras Endocrinol Metabol. 2011;55(9):701-7. 22231973 10.1590/s0004-27302011000900006

[48] Pabst R Tschernig T. Lymphocytes in the lung: an often neglected cell. Numbers, characterization and compartmentalization. Anat Embryol. 1995;192(4):293-9. 10.1007/BF00710098 8554162

[49] Beck FW Kaplan J Fine N. Decreased expression of CD73 (ecto-5’-nucleotidase) in the CD8+ subset is associated with zinc deficiency in human patients. J Lab Clin Med. 1997;130(2):147-56. 9280142 10.1016/s0022-2143(97)90091-3

[50] Maywald M Meurer S Weiskirchen R. Zinc supplementation augments TGF-β1 dependent regulatory T cell induction. Mol Nutr Food Res. 2017;61(3):1600493. 10.1002/mnfr.201600493 27794192

[51] Da M Chen L Enk A. The multifaceted actions of CD73 during development and suppressive actions of regulatory T cells. Review. Front Immunol. 2022;13:914799. 35711418 10.3389/fimmu.2022.914799 PMC9197450

[52] Tharp KM Kersten K Maller O. Tumor-associated macrophages restrict CD8(+) T cell function through collagen deposition and metabolic reprogramming of the breast cancer microenvironment. Nat Cancer. 2024;5(7):1045-62. 38831058 10.1038/s43018-024-00775-4 PMC12204312

[53] Krone A Fu Y Schreiber S. Ionic mitigation of CD4+ T cell metabolic fitness, Th1 central nervous system autoimmunity and Th2 asthmatic airway inflammation by therapeutic zinc. Sci Rep. 2022;12(1):1943. 35121767 10.1038/s41598-022-04827-6 PMC8816938

[54] Elmadfa I Meyer AL. The role of the status of selected micronutrients in shaping the immune function. Endocr Metab Immune Disord Drug Targets. 2019;19(8):1100-15. 31142256 10.2174/1871530319666190529101816 PMC7360912

[55] Lue C Kiyono H McGhee JR. Recombinant human interleukin 6 (rhIL-6) promotes the terminal differentiation of in vivo-activated human B cells into antibody-secreting cells. Cell Immunol. 1991;132(2):423-32. 1988161 10.1016/0008-8749(91)90039-e

[56] Fraker PJ King LE. Reprogramming of the immune system during zinc deficiency. Annu Rev Nutr. 2004;24:277-98. 15189122 10.1146/annurev.nutr.24.012003.132454

[57] Wang L Zhou M Kong X. Specific targeting of STAT3 in B cells suppresses progression of B cell lymphoma. Int J Mol Sci. 2023;24(17):13666. 37686472 10.3390/ijms241713666 PMC10563066

[58] Truong-Tran AQ Carter J Ruffin RE. The role of zinc in caspase activation and apoptotic cell death. Biometals. 2001;14(3-4):315-30. 11831462 10.1023/a:1012993017026

[59] Hojyo S Miyai T Fujishiro H. Zinc transporter SLC39A10/ZIP10 controls humoral immunity by modulating B-cell receptor signal strength. Proc Natl Acad Sci U S A. 2014;111(32):11786-91. 25074919 10.1073/pnas.1323557111 PMC4136588

[60] Caro-Maldonado A Wang R Nichols AG. Metabolic reprogramming is required for antibody production that is suppressed in anergic but exaggerated in chronically BAFF-exposed B cells. J Immunol. 2014;192(8):3626-36. 24616478 10.4049/jimmunol.1302062 PMC3984038

[61] Sadeghsoltani F Mohammadzadeh I Safari M-M. Zinc and respiratory viral infections: important trace element in anti-viral response and immune regulation. Biol Trace Elem Res. 2022;200(6):2556-71. 34368933 10.1007/s12011-021-02859-z PMC8349606

[62] Lin RJ Huang CH Liu PC. Zinc finger protein ZFP36L1 inhibits influenza A virus through translational repression by targeting HA, M and NS RNA transcripts. Nucleic Acids Res. 2020;48(13):7371-84. 32556261 10.1093/nar/gkaa458 PMC7367194

[63] Khan NA Singla M Samal S. Respiratory syncytial virus-induced oxidative stress leads to an increase in labile zinc pools in lung epithelial cells. mSphere. 2020;5(3):e00447-20. 32461278 10.1128/mSphere.00447-20 PMC7253603

[64] Tabatabaeizadeh SA. Zinc supplementation and COVID-19 mortality: a meta-analysis. Eur J Med Res. 2022;27(1):70. 35599332 10.1186/s40001-022-00694-z PMC9125011

[65] World Health Organization. COVID-19 dashboard: deaths. Accessed November 16, 2024. https://data.who.int/dashboards/covid19/deaths

[66] Fan L Cui Y Liu Z. Zinc and selenium status in coronavirus disease 2019. Biometals. 2023;36(5):929-41. 37079168 10.1007/s10534-023-00501-0 PMC10116102

[67] te Velthuis AJW van den Worm SHE Sims AC. Zn2+ Inhibits coronavirus and arterivirus RNA polymerase activity in vitro and zinc ionophores block the replication of these viruses in cell culture. PLoS Pathog. 2010;6(11):e1001176. 21079686 10.1371/journal.ppat.1001176 PMC2973827

[68] Polak Y Speth RC. Metabolism of angiotensin peptides by angiotensin converting enzyme 2 (ACE2) and analysis of the effect of excess zinc on ACE2 enzymatic activity. Peptides. 2021;137:170477. 33400951 10.1016/j.peptides.2020.170477 PMC7887068

[69] Costello LC Fenselau CC Franklin RB. Evidence for operation of the direct zinc ligand exchange mechanism for trafficking, transport, and reactivity of zinc in mammalian cells. J Inorg Biochem. 2011;105(5):589-99. 21440525 10.1016/j.jinorgbio.2011.02.002 PMC3081963

[70] Lee MC Chen YK Tsai-Wu JJ. Zinc supplementation augments the suppressive effects of repurposed NF-κB inhibitors on ACE2 expression in human lung cell lines. Life Sci. 2021;280:119752. 34171382 10.1016/j.lfs.2021.119752 PMC8219909

[71] Truong-Tran AQ Ruffin RE Foster PS. Altered zinc homeostasis and caspase-3 activity in murine allergic airway inflammation. Am J Respir Cell Mol Biol. 2002;27(3):286-96. 12204890 10.1165/rcmb.2001-0014OC

[72] Dufner-Beattie J Langmade SJ Wang F. Structure, function, and regulation of a subfamily of mouse zinc transporter genes. J Biol Chem. 2003;278(50):50142-50. 14525987 10.1074/jbc.M304163200

[73] Takagishi T Hara T Fukada T. Recent advances in the role of SLC39A/ZIP zinc transporters in vivo. Int J Mol Sci. 2017;18(12):2708. 29236063 10.3390/ijms18122708 PMC5751309

[74] Stewart-Knox BJ Simpson EE Parr H. Zinc status and taste acuity in older Europeans: the ZENITH study. Eur J Clin Nutr. 2005;59(Suppl 2):S31-6. 10.1038/sj.ejcn.1602295 16254578

[75] ter Borg S Verlaan S Hemsworth J. Micronutrient intakes and potential inadequacies of community-dwelling older adults: a systematic review. Br J Nutr. 2015;113(8):1195-206. 25822905 10.1017/S0007114515000203 PMC4531469

[76] Giacconi R Malavolta M Costarelli L. Comparison of intracellular zinc signals in nonadherent lymphocytes from young-adult and elderly donors: role of zinc transporters (Zip family) and proinflammatory cytokines. J Nutr Biochem. 2012;23(10):1256-63. 22209006 10.1016/j.jnutbio.2011.07.005

[77] Hara T Takeda T-A Takagishi T. Physiological roles of zinc transporters: molecular and genetic importance in zinc homeostasis. J Physiol Sci. 2017;67(2):283-301. 28130681 10.1007/s12576-017-0521-4 PMC10717645

[78] Wong CP Magnusson KR Ho E. Increased inflammatory response in aged mice is associated with age-related zinc deficiency and zinc transporter dysregulation. J Nutr Biochem. 2013;24(1):353-9. 22981370 10.1016/j.jnutbio.2012.07.005 PMC3586240

[79] Cabrera AJ. Zinc, aging, and immunosenescence: an overview. Pathobiol Aging Age Relat Dis. 2015;5:25592. 25661703 10.3402/pba.v5.25592 PMC4321209

[80] Mocchegiani E Malavolta M Muti E. Zinc, metallothioneins and longevity: interrelationships with niacin and selenium. Curr Pharm Des. 2008;14(26):2719-32. 18991691 10.2174/138161208786264188

[81] National Institutes of Health office of Dietary supplements. Zinc - Health Professional Fact Sheet. Updated March 26, 2022. Accessed July 11, 2024. https://ods.od.nih.gov/factsheets/Zinc-HealthProfessional/#en24

[82] Babaali E Rahmdel S Berizi E. Dietary intakes of zinc, copper, magnesium, calcium, phosphorus, and sodium by the general adult population aged 20-50 years in Shiraz, Iran: a total diet study approach. Nutrients. 2020;12(11):3370. 33139663 10.3390/nu12113370 PMC7693320

[83] Roohani N Hurrell R Kelishadi R. Zinc and its importance for human health: an integrative review. J Res Med Sci. 2013;18(2):144-57. 23914218 PMC3724376

